# The Influence of Chemical Structure on the Electronic Structure of Propylene Oxide

**DOI:** 10.3390/ijms262311729

**Published:** 2025-12-03

**Authors:** David G. Matalon, Kate L. Nixon, Darryl B. Jones

**Affiliations:** 1 School of Life, Health & Chemical Sciences, The Open University, Walton Hall, Milton Keynes MK7 6AA, UK; david.matalon@open.ac.uk; 2 College of Science and Engineering, Flinders University, GPO Box 2100, Adelaide, SA 5001, Australia

**Keywords:** propylene oxide, electron momentum spectroscopy, orbital electronic structure

## Abstract

Propylene oxide is the first and only chiral molecule to have been observed in the interstellar medium. Given the mechanisms for forming chiral species, which are important for astrobiology in understanding the origins of life, we report here an experimental and theoretical investigation into the electronic structure of propylene oxide and its evolution from the methylation and epoxidation of ethene. Here, electron momentum spectroscopy is used as an orbital-imaging technique to probe experimental orbital momentum distributions. These are directly compared with theoretical orbital momentum distributions calculated at the equilibrium geometry, and those calculated by considering the vibrational motion of the propylene oxide target. This allows us to identify which molecular orbitals are sensitive to specific vibrational normal modes, thereby facilitating understanding and controlling chemical reactivity. By extending our investigation to include intermediate species along the evolution of ethene through methylation and epoxidation, we can develop an understanding of how the orbital electronic structure evolves through this series of important chemicals.

## 1. Introduction

**Figure 1:**
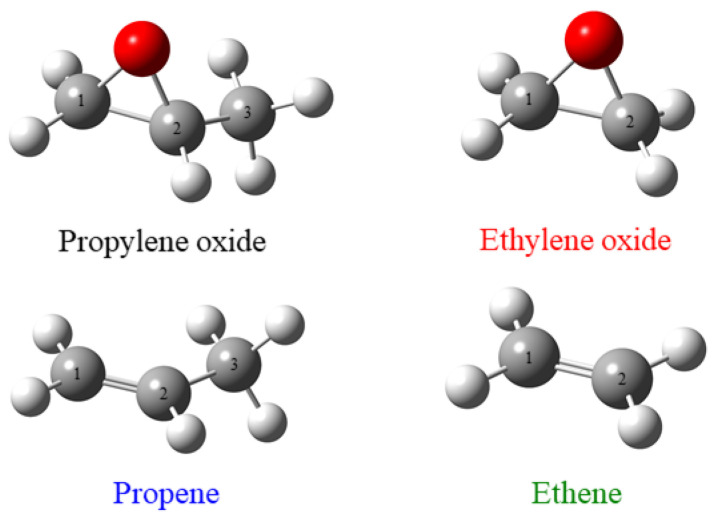
The chemical structures of propylene oxide (PO), ethylene oxide (EO), propene (PE), and ethene (EE). Here, carbon atoms are labeled to assist with discussions in the text.

The smooth evolution and conservation of orbital symmetry in chemical reactions has formed a basis for understanding stereochemical reactions [1]. Stereochemistry, and, in particular, chirality, strongly influence the biological activity of molecular systems. This has posed significant questions about the origin of life, and the preference for some homochirality in a heterochiral world [2]. These questions still remain [3] and influence many aspects of current investigation, from symmetries in fundamental particle physics [4] to the mechanisms of biological aging [5]. Recently, propylene oxide (C_3_OH_6_, see Figure 1) has been observed in the interstellar medium [6]. As a chiral molecular system, it provides a potential target for characterizing the presence of enantiomeric excess in interstellar regions, which could serve as a source of homochirality within planetary systems that may form. Here, significant questions remain regarding the mechanisms of synthesis of complex organic molecules in the interstellar medium (ISM). Specifically, it is unclear whether they form in the gas phase or in a condensed state on or within chondrites or icy grains [7]. This results from the molecules being detectable only through gas-phase rovibrational spectroscopy. Furthermore, current techniques cannot determine whether there is any enantiomeric excess in the ISM [6]. The observation of propylene oxide under such harsh environmental conditions found in the interstellar medium has since prompted significant experimental and theoretical interest. These studies include spectroscopic characterization [8,9,10] and studies of ionization and fragmentation dynamics [11,12,13].

Propylene oxide is also an industrially important chemical, as a precursor to polyurethane [14,15]. Here, propylene oxide has been traditionally formed through a synthetic route requiring hydrogen peroxide, whose production requires significant energy and generates waste [16]. As such, there have been increasing efforts to reduce emissions and waste chemicals formed during its production, and also improve the cost/energy efficiency of the processes involved [15]. Propylene oxide is also formed as an important reactive intermediate in combustion mechanisms [17], and with its reactivity, is also prone to further oxidation [15]. It is therefore desirable to fully understand propylene oxide’s electronic structure, as this may assist in identifying the mechanisms of its production in harsh environments and enable new synthetic strategies to be developed that may improve its industrial production.

Electron momentum spectroscopy (EMS) [18,19] is an experimental technique observing the kinematically complete electron impact ionization reaction of an at rest target molecule *T*:

(1) $$e_{0}^{-}\left(E_{0},p_{0}\right)+T\to e_{Sc}^{-}\left(E_{Sc},p_{Sc}\right)+e_{Ej}^{-}\left(E_{Ej},p_{Ej}\right)+T_{i}^{+}\left(\varepsilon_{i},p_{recoil}\right).$$

Here, $e_{j}^{-}\left(E_{j},p_{j}\right)$ is an electron with energy ($E_{j}$) and momentum ($p_{j}$), where the subscript *j* = 0, *Sc*, *Ej* corresponds to the incident, fast scattered, or slow ejected electron, respectively. Here $\varepsilon_{i}$ is the energy required to remove the bound electron from the neutral target and produce the $T_{i}^{+}$ ion in the *i*th state, which has a recoil momentum following the ionization, $p_{recoil}$. Through the conservation of energy and momentum in the collision,

(2) $$E_{0}=E_{Sc}+E_{Ej}+\varepsilon_{i}$$

(3) $$p_{0}=p_{Sc}+p_{Ej}+p_{recoil}.$$

By choosing experimental conditions that mimic a free electron collision, the recoil momentum of the residual ion is equal and opposite to the momentum, p, of the electron at the instant it was ionized ($p_{recoil}$ = −p). Further, by considering this ionization process of a randomly oriented molecule within the plane wave impulse approximation (PWIA), the cross section describing the probability of this ionization event occurring is then proportional to the spherical average of the square of the Dyson orbital:

(4) $$\sigma_{i}\left(p\right)\propto \int d\Omega {\left|\left\langle p\Psi_{i}^{+}|\Psi_{0}\right\rangle \right|}^{2}.$$

Within a target Hartree–Fock or Kohn–Sham approximation [18,20], the Dyson orbital can be approximated by the ionized ground state orbital $\phi_{j}(p)$ through the introduction of a spectroscopic factor, $S_{j}^{\left(i\right)}$:

(5) $$\left\langle p\Psi_{i}^{+}|\Psi_{0}\right\rangle =\sqrt{S_{j}^{\left(i\right)}}\phi_{j}\left(p\right).$$

Here, the spectroscopic factors describe the square of the amplitude of the *j*^th^ orbital’s one-hole configuration in the *i*^th^ final ion state, such that the spectroscopic factors satisfy the spectroscopic sum rule:

(6) $$\sum_{i}S_{j}^{\left(i\right)}=1.$$

As such, EMS provides a powerful method for investigating the molecular orbitals and electronic structure of molecular systems.

With this in mind, we have undertaken an experimental and theoretical investigation into the electronic structure of propylene oxide. Here, we employ electron momentum spectroscopy as an orbital imaging technique to identify and characterize propylene oxide’s orbital structure. Further, we compare the structure of propylene oxide with those of chemically similar units (ethylene oxide, ethene, and propylene, see Figure 1). These structurally similar systems are considered as we postulate that the epoxide mimics the CC double bond, with epoxides able to be formed through an epoxidation of an alkene by a peroxy acid [21]. Further, we also consider how the addition of the methyl group influences the structure. This work aims to provide insights into how the orbital structure evolves as chemical adducts increase the molecular complexity. With this work, we hope to unlock processes that may explain how complex molecular structures are generated under the harsh conditions of the interstellar medium and also assist us in improving synthetic chemical methods. Here, the comparison is enabled as ethylene [22,23,24], propene [25,26] and ethylene oxide [27,28], have all previously been investigated using the EMS technique to support our independent theoretical analysis across the chemical series. This work also builds on existing attempts to assign orbital ordering of substituted oxiranes using photoelectron spectroscopy [29]. Here, EMS, probing the electron momentum dependence of the ionization channel, provides further insight into the orbital electronic structure, enabling a more thorough understanding of how the electronic structure evolves with increasing chemical complexity.

## 2. Results and Discussion

### 2.1. Binding Energy Spectra

**Figure 2:**
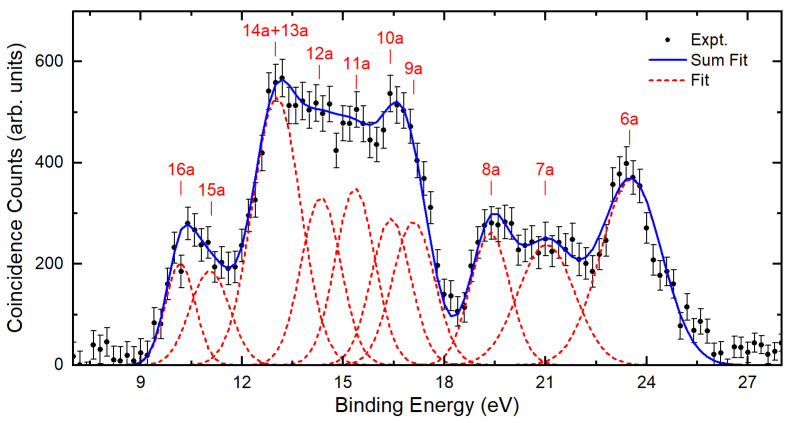
The experimentally measured total binding energy spectrum of propylene oxide is obtained by summing up the binding energy spectra at each ejected analyzer angle. Also shown are the fitting functions used to deconvolve the spectra and extract the momentum distributions for each feature, and their sum.

In Figure 2, we present the total binding energy spectrum for propylene oxide that was obtained by summing the binding energy spectra recorded at each polar ejected angle, with the table of ionization energies and orbital assignments shown in Table 1. Here, we observe a spectrum with features consistent with previously measured photoelectron spectra [29,30]. The present SAC-CI and OVGF calculations give energies consistent with the broad features seen in both the present binding energy spectrum and the photoelectron spectrum (PES), and the calculated pole strength for the outer valence states, with pole strengths (PS) > 0.8 supporting a single particle picture of ionization. In Table 1, we also include the ionization energies obtained from the ωB97X-D/aug-cc-pVTZ calculation. Here, we observe that DFT methods are generally less reliable at predicting ionization energies than the OVGF and SAC-CI methods. The present EMS binding energy spectrum is deconvoluted using Gaussian fitting functions, as shown in Figure 2, to obtain the momentum distributions (MDs) for each resolvable ionization channel. Here, the fitting parameters (energies and widths) were initially selected based on the spectral widths and energies of features reported in the PES and convoluting the widths with our instrumental energy resolution. Some variation from the initial parameters was allowed to enable the best fit to the observed experimental binding energy spectrum. Experimental MDs are then obtained through this deconvolution procedure for the binding-energy spectrum measured at each polar angle.

### 2.2. Experimental and Theoretical Momentum Distributions

**Figure 3:**
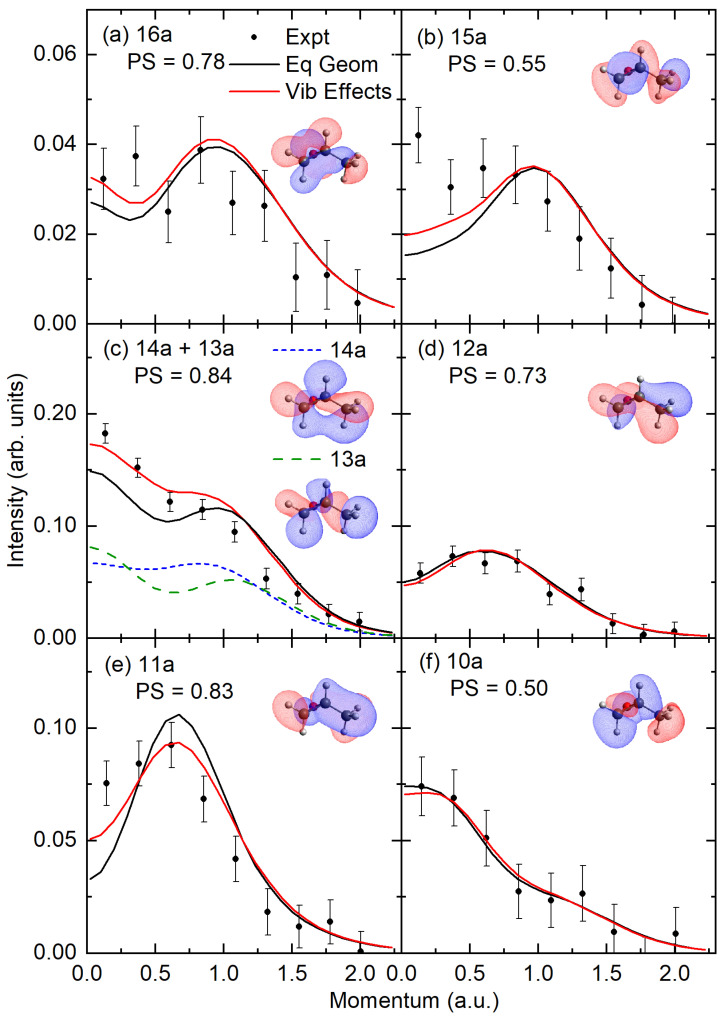
The experimental and theoretical MDs. Experimental data are globally normalized to the theoretical MDs, assuming an experimental PS of 0.84 for the strongest unresolved 14a + 13a orbitals. The theoretical MDs are then rescaled to obtain PSs for the remaining ionized orbitals. Here, the PSs are applied to each orbital in an unresolved sum, the equilibrium geometry MD, and the MD incorporating vibrational effects. See legends and text for further details.

**Figure 4:**
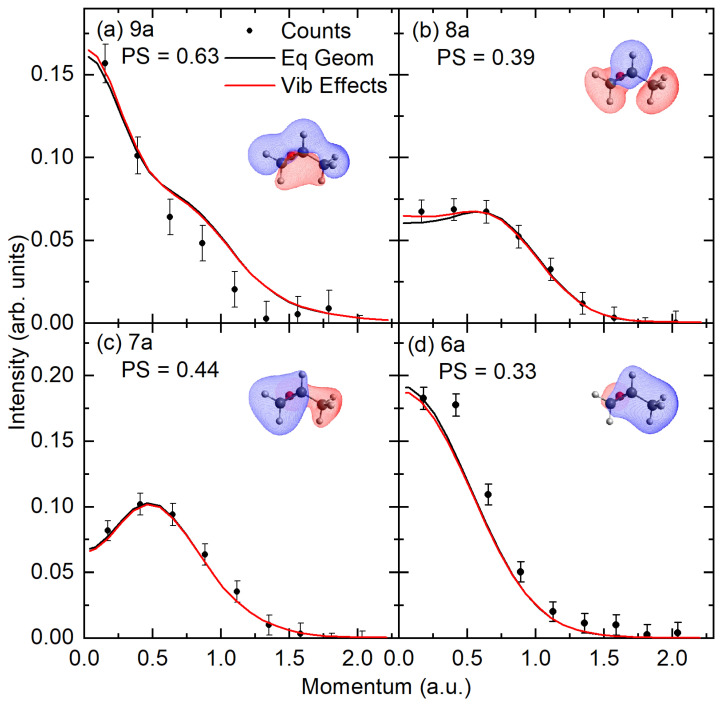
The experimental and theoretical MDs (continued). Experimental data are globally normalized to the theoretical MDs, assuming an experimental PS of 0.84 for the strongest unresolved 14a + 13a orbitals. The theoretical MDs are then rescaled to obtain PSs for the remaining ionized orbitals. Here, the PSs are applied to each orbital in an unresolved sum, the equilibrium geometry MD, and the MD incorporating vibrational effects. See legends and text for further details.

Here, the experimental and theoretical MDs are presented in Figure 3 and Figure 4. The theoretically presented MDs have been calculated at both the equilibrium geometry and with a correction for nuclear vibrational motion. In this figure, the common intensity scale is set by normalizing the experimental data to the unresolved sum of the 14a + 13a molecular orbitals, with the pole strengths for both MOs in this unresolved sum being 0.84 at the SAC-CI level. Here, the 14a and 13a molecular orbitals were chosen for normalization as they have the largest pole strengths from the SAC-CI calculations, and any orbital mixing is restricted to the ionized orbitals present within the unresolved band. Here, we note that although the HOMO is typically used for such a procedure, the present SAC-CI calculations suggest that the HOMO (16a) and NHOMO (15a) may both contribute to the first two ionization bands, which can be energetically resolved. Subsequently, the pole strengths for the other valence molecular orbitals are obtained using a least-squares fitting procedure to determine the scaling factor for the theoretical distributions that best reproduce the experimental data for the individual experimental features.

Here, we can observe that the shapes of the experimental MDs are in reasonable agreement with the orbital MDs after the vibrational effects have been included in the theoretical MDs. The vibrational effects most strongly influence the shape of the MDs in the unresolved 14a + 13a summed orbitals and the 11a orbital. In the summed 14a + 13a distribution, the vibrational effects lead to an increase in the intensity of the MD in the low momentum region, with the major increase stemming from the v_1_ (203 cm^−1^) normal mode which relates to the methyl rotation, the epoxide CH stretching modes, v_21_ (3119 cm^−1^) and v_24_ (3192 cm^−1^), and the CC methyl/epoxide connection stretching/CH bending motion normal mode v_15_ (1456 cm^−1^). Here, as both the 14a and 13a orbitals involve some coupling between the methyl group and the epoxide, it is not surprising that the orbital displays some sensitivity to the methyl rotation and CH-stretching/bending vibrational motions.

In the case of the 11a orbital, the vibrational motion again increases the intensity in the low momentum region but decreases the intensity at intermediate momenta (~0.6–1.0 a.u). In this case, the CC stretch joining the methyl (C_3_) and epoxide carbons (C_2_) v_7_ (1003 cm^−1^) and the methyl (C_3_) CH bending v_16_ (1489 cm^−1^)/v_17_ (1504 cm^−1^) vibrations increase the low momentum intensity, with these and many other modes collectively reducing the intensity at the intermediate momenta. These variations arise from the orbital having a significant π-like interaction coupling the methyl and epoxy carbon atoms, with additional contributions from the H-atoms.

In all other momentum distributions, the vibrational motion does not substantially alter the profile from that calculated at the equilibrium geometry. Further, we typically see good shape agreement between the experimental distribution and the theoretical distributions incorporating vibrational effects to within experimental uncertainty. Here, the determined pole strength (PS) in the outer valence region is typically in good agreement with the single-ionization picture, as supported by the SAC-CI/OVGF calculations, with experimental values above 0.73 for all but the 15a orbital, which has PS = 0.55. This value for the 15a orbital seems surprisingly low, as the 15a orbital is the next highest occupied molecular orbital (NHOMO), and the calculated PS for this orbital is consistent with the other outer valence orbital, being 0.84 and 0.91 at the SAC-CI and OVGF levels, respectively. As the SAC-CI theory suggests that there may be a mixing of the orbital contributions between the HOMO/NHOMO pair in the dominant configurations of the first two ion states, we attempted to sum the experimental HOMO and NHOMO but found no significant improvement in the interpretation of the experimental orbital momentum profiles. Upon investigation, we found that this behavior is not unique to propylene oxide, with the HOMO of propene (2a′′) orbital having a pole strength that is 20% lower than the other outer valence orbitals [26], as discussed further below.

The reasonable agreement observed between most of the experimental momentum profiles and those calculated theoretically enables us to use the theoretical calculations with confidence to evaluate the electronic structural evolution of ethene through methylation and epoxidation.

### 2.3. Orbital Correlations

**Figure 5:**
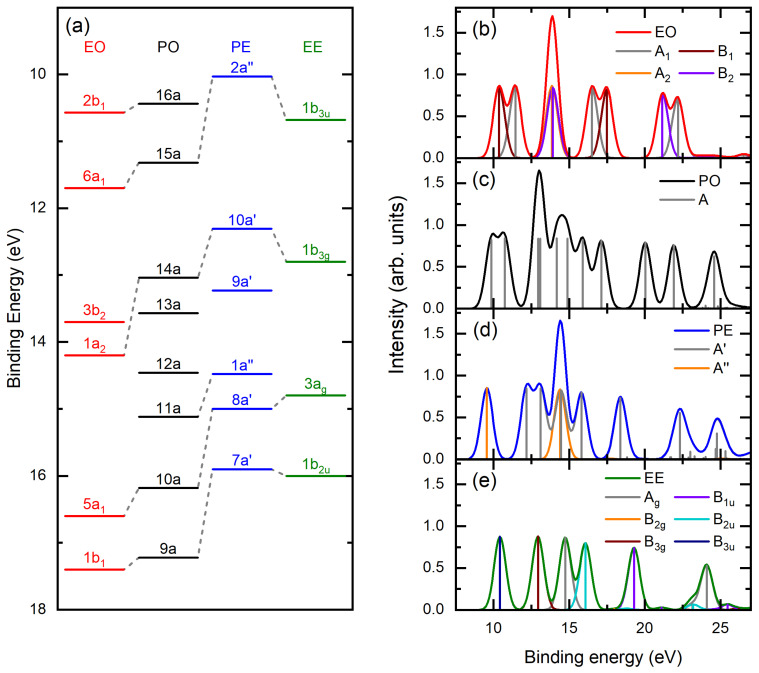
(**a**) Orbital correlation diagram based on reported photoelectron ionization energies [29,30,31,32] and simulated theoretical binding energy spectra for (**b**) EO, (**c**) PO, (**d**) PE, and (**e**) EE, assuming a FWHM of 0.9 eV to closely mimic the present instrumental resolution. Also shown are contributions to the spectra from symmetry manifolds and monopoles for states within those symmetry manifolds. See text and legends for further details.

Based on the available PES for propene [31], ethene [30,32], propylene oxide [29], ethylene oxide [29], and the presently simulated binding energy spectra obtained through SAC-CI calculations, we assign orbital correlations in Figure 5.

**Figure 6:**
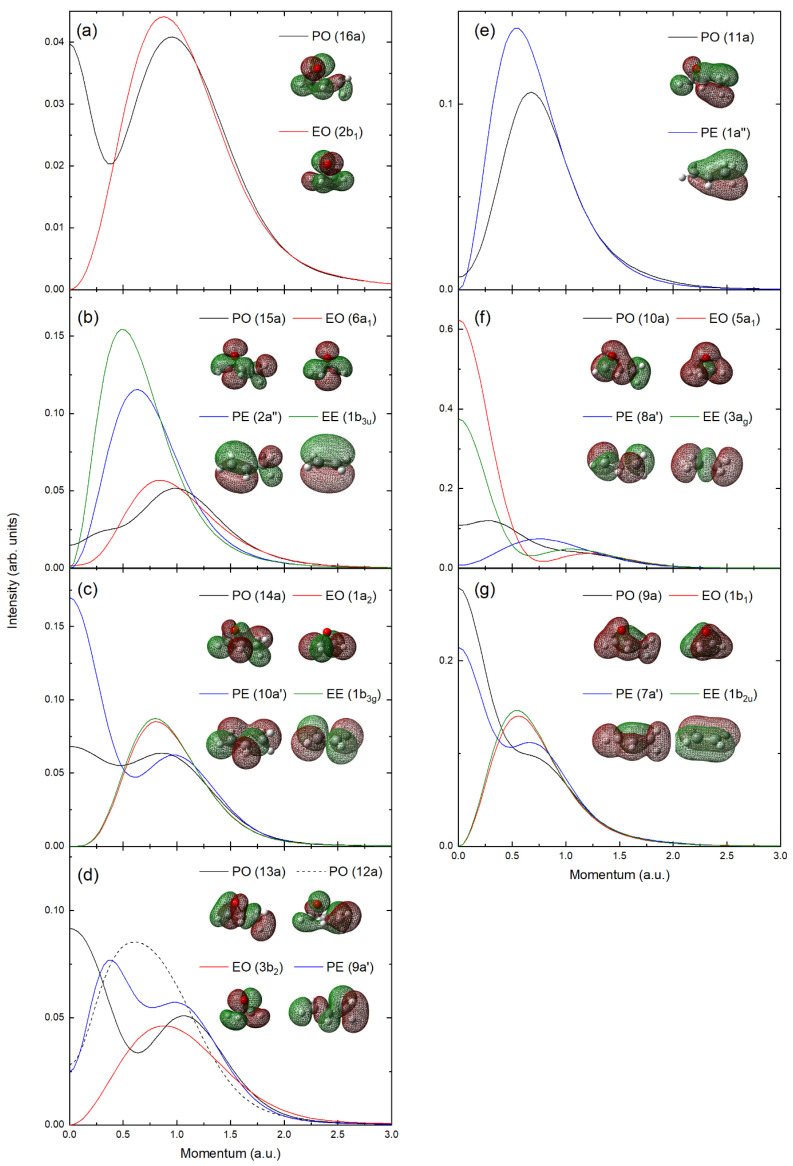
Moment distributions and orbital representations for EO, PO, PE, and EE. (**a**) PO (16a) and EO (2b_1_). (**b**) PO (15a), EO (6a_1_), PE (2a′′) and EE (1b_3u_). (**c**) PO (14a), EO (1a_2_), PE (10a′) and EE (1b_3g_). (**d**) PO (13a), PO (12a), EO (3b_2_) and PE (9a′). (**e**) PO (11a) and PE (1a′′). (**f**) PO (10a), EO (5a_1_), PE (8a′) and EE (3a_g_). (**g**) PO (9a), EO (1b_1_), PE (7a′) and EE (1b_2u_). See legends and text for further details.

The first correlated orbitals are the PO (16a) and EO (2b_1_), with the MDs and orbital representations being presented in Figure 6a. Here, we observe that these orbitals are typically described in PES spectra as a non-bonding O(2p) contribution, which couples to asymmetric C(2p) and H(s) contributions about the epoxy symmetry plane. In this case, we see that the MD has a dominant p-like feature giving rise to a peak at ~1.0 a.u. However, the methylation of the epoxide group allows the C(2p) contributions to mix to form a CC σ-like bonding contribution that appears to generate a significant MD contribution at ~0 a.u for PO.

In Figure 6b, we present the MDs and orbital representations of the PO (15a), EO (6a_1_), PE (2a″), and EE (1b_3u_). We describe these orbitals as the out-of-plane H_2_CCHX π-bonding contribution. In the EE, the CC double bond gives a π-bonding orbital from the overlapping of the C(2p) atomic orbitals. This characteristic orbital behavior is only slightly disrupted by methylation in PE, with the PE MD having a similar distribution but shifting to higher momentum values. The epoxidation distorts the H2CCHX frame, but the dominant C(2p) atomic contributions to the orbital character remain. Here, they rotate with the distortion and mix with O(2p) orbitals to stabilize the highly strained epoxide ring. This reduces the intensity at the MD maxima but slightly increases the MD intensity at higher momentum compared to EE/PE. In the case of PO, the methylation also couples these p-orbital interactions into a σ-like bonding contribution to give an additional low-momentum feature in the MD. Here, we also observe that methylation in PO shifts the peak of the orbital MD to a higher momentum than that for EO, similar to that observed between PE and EE. Note that it is for this orbital that we observe the lower PS, and that it has also been observed in PE. In the cases of EO and EE, the reported EMS studies [22,24,27] do not explicitly report PSs, but the results, when compared with our present calculations for EO and EE, suggest no significant alteration of the PS from the other outer valence orbitals. The variation in the pole strength, therefore, seems to be related to the methylation. Interestingly, the HOMO of acetone, a structural isomer of propylene oxide with two methyl rotors, has a momentum distribution that cannot be fully reproduced by theory [33,34]. Also dimethyl ether [35] and dimethylsulfide [36] have similar reproduction issues with their experimental MDs. It may be that current quantum chemical models do not adequately couple the interactions of the methyl group(s) with other functional groups, so that their experimental MDs cannot be fully reproduced through theory. Under the present condition, where we have a somewhat low ejected electron energy (101 eV), we may have a restricted ability for quantitative extraction of the pole strengths through not fully reaching the limit required for the PWIA. Contrary to this point, the experiments on PE were performed at an impact energy of ~1200 eV in a symmetric coplanar geometry where the PWIA limit should be reached. As such, further experiments testing the validity of the PWIA for these systems may be required to resolve this point.

The MDs and orbital representations for PO (14a), EO (1a_2_), PE (10a′), and EE (1b_3g_) are presented in Figure 6c. For EO and EE, these orbitals constitute an asymmetric contribution of H(1s) atomic contributions with 4 lobes mimicking a CC π*-orbital. This gives the orbital MDs a strong p-like distribution, which is largely unchanged between EE and EO. Here, methylation facilitates a CC σ-bonding interaction, which enhances the MD in the low-momentum region of PE. In PO, the additional distortion still enables this CC σ-bonding interaction, but it does not increase the MD as significantly as in PE in the low-momentum region.

In the paper of McAlduff and Houk [29], orbital correlations of the four outermost molecular orbitals across the series of substituted oxiranes are used to assign and assess the photoelectron spectra. In looking at the orbital correlation diagram, we agree with the interpretation of the two outermost orbitals but are uncertain about the order of the innermost of these four molecular orbitals, where it is reported that the orbital ordering does not change from EO to PO. In EO, photoelectron spectroscopy assignments place the 3b_2_ with lower binding energy than the 1a_2_ orbital, with this assignment being made through comparison of He(I) and He(II) spectral intensities [37]. Our present SAC-CI calculations invert this energetic ordering in EO, but the states are too closely spaced in energy, so previous EMS studies on EO could not resolve these features [27,28]. These MOs are energetically reordered in PO in all of our calculations, with the EO (1a_2_) correlating to the PO (14a), contrary to previous PES assignments [29]. In Figure 6d, we present the momentum distributions and orbital representations for EO (3b_2_), PO (13a and 12a), and PE (9a′). In EO, this orbital has an O(2p) contribution that with the high molecular orbital symmetry couples with the H-atom contributions, and the momentum profile has a characteristic p-like shape. In the PO case for the 13a/12a orbitals, the methylation breaks this symmetry, and the O contributions rotate and more extensively couple through the CH network. This is evident in the momentum distribution where both orbital MDs have substantial intensity in the low-momentum region (*p* < 0.5 a.u). This coupling is also seen in PE (9a′). As such, both PO (13a/12a) orbitals have a mixing of the closely lying in energy orbital characters of EO (3b_2_) and PE (9a′) orbitals, and a unique correspondence of orbital correlation across the systems is problematic, contrary to previous reports.

Here, in Figure 6e, the PO (11a) and PE (1a′′) are attributed to the methylation, with both orbitals giving characteristic momentum distributions relating to the out of CC plane π-orbital, although the distortion relating to the epoxidation does alter the MD intensities (see Figure 6e). The energetic ordering of the PE 1a′′ and 8a′ has been a subject of debate, with original PES assignments preferring 1a′′ to have a lower ionization potential than 8a′, but Ning et al. [25] and Bawagan et al. [31] revised this ordering based on OVGF and MRSDCI calculations. Unfortunately, the states were unresolvable in the EMS spectrum, so it cannot resolve the discrepancy. The present DFT and SAC-CI calculations favor the present ordering, although the states are always closely lying in energy. Here, we find that both the PO (11a) and PE (1a″) orbitals display a characteristic p-like MD, attributable to the local π-like bonding between the CC bond formed through the methylation. Here, the PO (10a) and PE (8a′) orbitals also have a p-like momentum profile, arising from a network of C(2p) contributions through the C-backbone. In the case of PO, this MD gains an increased s-like contribution in the MD through the O-atom participation from outside the CCC plane.

In Figure 6f, the PO (10a), EO (5a_1_), PE (8a′), and EE (3a_g_) momentum distributions are presented. Here, EE and EO have a prominent CC σ bonding character that is enhanced in the low momentum region by symmetry-allowed participation of the H s-orbitals. In PO and PE, the methylation, destroying the high symmetry, twists these orbital contributions out of the CC bond axis to inhibit some H-atom contribution and alter the momentum profile shapes.

Lastly, in Figure 6g, we can observe the momentum distributions for the PO (9a), EO (1b_1_), PE (7a′), and EE (1b_2u_) orbitals. These orbitals involve an out of CC(O)-plane π-like contribution with participation from the H atoms in EE (PE), and this is reflected in the MD shape. The methylation results in curvature of this CCCO plane, increasing the asymmetry in the atomic contributions to produce a strong MD intensity at low momenta, giving the MDs for these PO and PE orbitals very different shapes from their unmethylated counterpart orbitals in EO and EE.

## 3. Materials and Methods

An experimental EMS investigation was performed at the Open University using an apparatus that has been previously described in detail [38]. Here, an asymmetric coplanar kinematic geometry is used. A beam of incident electrons with energy $E_{0}=821+\varepsilon_{i}\;$eV is produced using an electron gun comprising a thoriated tungsten filament and two three-element cylindrical lenses. Scattered and ejected electrons are then detected by electron analyzers mounted on independently rotatable turntables. Each analyzer is composed of cylindrical lens stacks to decelerate electrons leaving the collision region and transport them to a hemispherical selector. The selector disperses the electrons based on their kinetic energy, with those electrons traversing the hemisphere being detected using a position-sensitive detector comprising a pair of microchannel plates and a resistive anode. Here, the fast scattered electron is detected with energy $E_{1}\sim 720$ eV at a polar angle of 20.5°, while the ejected electron is detected with energy $E_{2}\sim 101$ eV as the analyzer is moved over a range of polar angles between 70 and 110°. Note that all angles are measured with respect to the incident beam direction. Here, the true coincident electron count rate at each accessed binding energy is observed while scanning over a range of polar angles. Note that scanning over polar angles is equivalent to scanning over a range of recoil momenta magnitudes, allowing for experimental determination of an ionization channel’s momentum distribution (MD). Here, the coincidence energy resolution is 1.0 eV, and the momentum resolution is 0.28 a.u.

Propylene oxide (>99% assay, Sigma Aldrich (Gillingham, UK), 50:50 racemic mixture) as received was subjected to repeated freeze–pump–thaw cycles to remove dissolved gases. As a temperature-sensitive chemical (recommended storage between 2 and 8 °C to minimize polymerization) with a high vapor pressure (~600 mbar at ~300 K) [39], the liquid sample was stored in an ice bath at 0 °C during the measurements. Measurements were conducted by introducing sample vapor into the chamber, raising the base chamber pressure from 3 × 10^−7^ to 1.3 × 10^−6^ Torr.

For comparison to the experimental measurements, we obtain theoretical orbital MDs by performing a spherical average of Kohn-Sham orbitals obtained from quantum chemical calculations that have been transformed into the momentum space representation. These were carried out for the range of molecular targets using the HEMS program [40]. To further assess the role of nuclear motion on the MDs, we also extend this analysis following the procedure of Watanabe et al. [22,41] to consider how geometric distortion along each vibrational normal mode influences the obtained orbital MD. Here, the resultant orbital MD incorporating vibrational effects is evaluated as follows:

(7) $$M\left(p\right)=\rho_{f}\left(p,\;0\right)+\sum_{L}\left\langle \xi_{vL}|\rho_{f}\left(p,Q_{L}{\hat{q}}_{L}\right)-\rho_{f}\left(p,\;0\right)|\xi_{vL}\right\rangle .$$

Here $\rho_{f}\left(p,\;0\right)$ is the MD evaluated at the equilibrium geometry, while we have a correction term that is created through evaluating the difference in the distribution between the equilibrium geometry and the MD as the geometry is distorted along a normal vector, ${\hat{q}}_{L}$, for the *L*-th vibrational normal mode, with this correction term evaluated analytically based on the properties of the harmonic oscillator. By evaluating the vibrational correction in this way, we can then assess the role of each vibrational normal mode on the obtained MDs. The total vibrational correction is then evaluated as a sum of each vibrational mode’s contributions to the MD, assuming a Boltzmann population of the vibrational modes in the ground electronic state, assuming a room temperature (*T* = 25 °C), as the vapor from the sample reaches an equilibrium temperature within the apparatus prior to the collision region. Through this procedure, we obtain MDs obtained at the equilibrium geometry, and those incorporating the influence of vibration motion.

To obtain the required structural information about the molecular targets, we performed a range of quantum-chemical calculations to describe the electronic structure of the ground neutral and ionized states. Typically, geometries were optimized at the ωB97X-D/aug-cc-pVTZ level [42,43,44], with the optimum geometries being used for further frequency analysis and single point energy calculations at the same level of theory, and additional calculations using outer valence Green’s function (OVGF) methods [45] and symmetry-adapted cluster configuration interaction calculations (SAC-CI) [46] which can provide reliable ionization potentials to assist with the spectroscopic assignments. These calculations were performed using the Gaussian suite of programs [47,48], with the computational work supported by a high-performance computational environment [49]. These quantum chemical methods were selected as they provide the necessary balance between available computational resources and chemical accuracy. Lastly, in order to directly compare the present experimental and theoretical MDs, the calculated MDs have been convoluted with an experimental instrument response using an implementation of a Gaussian weighted planar grid method [50] that adequately reproduced the experimental momentum resolution of 0.28 a.u.

## 4. Conclusions

Here, we presented a joint experimental and theoretical study of the electronic structure of propylene oxide. The shapes of the experimental momentum profiles were well reproduced using theoretical calculations for the outer valence orbitals. Interestingly, we observed differences between the obtained spectroscopic factors for some orbitals and the theoretical pole strengths predicted. This may be due in part to the limitations of the PWIA under the present asymmetric kinematics with a slow ejected energy of 101 eV. Here, we also observed an unusually low PS for the propylene oxide 15a orbital, with this behavior being similar to that observed for the correlated 2a′′ orbital in propene [25,26], which was measured under conditions where the PWIA should be valid. By considering the orbital structure of other related species, ethylene, ethylene oxide, and propylene, we were able to understand how the orbital structure of ethylene evolves through methylation and epoxidation to that observed for propylene oxide. Though understanding how electronic structure evolves in chemically related systems, we are better placed to predict the formation of chemical species, the reactivity of a chemical target, and the ability of other species to recognize a molecular structure.

## Figures and Tables

**Table 1:** The experimental and theoretical ionization energies (IE) and spectroscopic assignments. ^#^ PS greater than 0.1; ^$^ The ordering and assignments is taken from the ωB97X-D calculation, and the 16a/15a ordering is reversed at the HF level.

Assignment ^$^	Present EMS	PES [30]	PES [29]	ωB97X-D/ aug-cc-pVTZ	OVGF/ aug-cc-pVTZ ^$^		SAC-CI/6-311G**		
	IE (eV)	IE (eV)	IE (eV)	IE (eV)	IE (eV)	PS	IE (eV)	PS ^#^	Dominant Configurations ^$^
16a	10.2	10.44	10.26	9.74	10.89	0.90	9.85	0.82	0.88(15a)^−1^ + 0.36(16a)^−1^
15a	11.1	11.32	11.23	10.51	11.16	0.91	10.73	0.84	−0.89(16a)^−1^ + 0.36(15a)^−1^
14a	13.0	13.04	12.88	12.32	13.36	0.91	12.93	0.84	0.95(14a)^−1^ + 0.17(13a)^−1^
13a	13.0	13.57	13.33	12.57	13.43	0.91	13.09	0.84	−0.95(13a)^−1^ + 0.17(14a)^−1^
12a	14.3	14.46		13.41	14.54	0.91	14.18	0.84	0.96(12a)^−1^
11a	15.4	15.12		14.08	15.19	0.91	14.87	0.84	−0.96(11a)^−1^
10a	16.4	16.18		15.25	16.17	0.90	15.89	0.82	−0.95(10a)^−1^
9a	17.1	17.22		16.41	17.41	0.90	17.12	0.81	−0.94(9a)^−1^
8a	19.4	19.65		18.88			20.03	0.79	0.94(8a)^−1^
7a	21.0			20.67			21.92	0.76	−0.92(7a)^−1^
6a	23.5			23.09			24.56	0.63	−0.83(6a)^−1^

## Data Availability

The original contributions presented in this study are included in the article. Further inquiries can be directed to the corresponding authors.

## Abbreviations

The following abbreviations are used in this manuscript:

DFT	density functional theory
EE	ethene
EMS	electron momentum spectroscopy
EO	ethylene oxide
HOMO	highest occupied molecular orbital
IE	ionization energy
ISM	interstellar medium
MD	momentum distribution
NHOMO	next highest occupied molecular orbital
OVGF	outer valence Green’s function
PE	propene
PES	photoelectron spectrum
PO	propylene oxide
PS	pole strength
PWIA	plane wave impulse approximation
SAC-CI	symmetry-adapted cluster configuration interaction
